# Technology transfer during the COVID-19 pandemic: report on the first face-to-face practical training course in Brazil

**DOI:** 10.1590/S2237-96222023000200017

**Published:** 2023-08-18

**Authors:** Fernanda Khouri Barreto, Luciane Amorim Santos, Marta Giovanetti, Vagner Fonseca, Flavia Aburjaile, Joscelio Aguiar Silva, Carla Freitas, Cassio Roberto Leonel Peterka, Jairo Mendez Rico, Maria Almiron, Carlos Frederico Campelo de Albuquerque e Melo, Luiz Carlos Júnior Alcântara

**Affiliations:** 1 Universidade Federal da Bahia, Instituto Multidisciplinar em Saúde, Vitória da Conquista, BA, Brazil; 2 Escola Bahiana de Medicina e Saúde Pública, Salvador, BA, Brazil; 3 Fundação Instituto Oswaldo Cruz, Laboratório de Mosquitos Vetores - Endossimbiontes e Interação Patógeno-Vetor, Belo Horizonte, MG, Brazil; 4 Organização Pan-Americana da Saúde, Organização Mundial da Saúde, Brasília, DF, Brazil; 5 Universidade Federal de Minas Gerais, Belo Horizonte, MG, Brazil; 6 Ministério da Saúde, Secretaria de Vigilância em Saúde e Ambiente, Coordenação-Geral das Arboviroses, Brasília, DF, Brazil; 7 Ministério da Saúde, Secretaria de Vigilância em Saúde e Ambiente, Coordenação-Geral de Laboratórios de Saúde Pública, Brasília, DF, Brazil; 8 Pan American Health Organization, Health Emergencies Department, Washington, DC, United States

**Keywords:** COVID-19, Pandemic, Professional Training, Health Human Resources Development, COVID-19, Pandemia, Capacitación Profesional, Capacitación de Recursos Humanos en Salud, Covid-19, Pandemia, Capacitação Profissional, Capacitação de Recursos Humanos em Saúde.

## Abstract

The objective of this report was to describe the first face-to-face course aimed at training public health professionals in performing real-time genomic surveillance during the pandemic period. Experience report on a theoretical-practical course focusing on genomic research and surveillance, including mobile sequencing technologies, bioinformatics, phylogenetics and epidemiological modeling. There were 162 participants in the event and it was the first major face-to-face training course conducted during the COVID-19 epidemic in Brazil. No cases of SARS-CoV-2 infection was detected among the participants at the end of the event, suggesting the safety and effectiveness of all safety measures adopted. The results of this experience suggest that it is possible to conduct professional training safely during pandemics, as long as all safety protocols are followed.

## Study contributions

**Main results:**

Technology transfer can take place at large events, as long as safety protocols are strictly enforced. It is important to disseminate, at these events, the concepts of the Responsible Research and Innovation (RRI).

**Implications for services:**

Face-to-face training course is fundamental for training public health professionals. Technology transfer between research institutions and health services results in updating and improving health system performance.

**Perspectives:**

Based on the success of the reported technology transfer, a new module will be incorporated into the next edition of VEME (Panama 2022), entitled Virus Evolution to Public Health Policy Makers.

## Resumo

O relato descrebeu o primeiro curso presencial visando capacitar profissionais de saúde pública na realização de vigilância genômica em tempo real, durante períodos pandêmicos. Relato de experiência sobre um curso teórico-prático com foco em pesquisa e vigilância genômica, incluindo tecnologias de sequenciamento móvel, bioinformática, filogenética e modelagem epidemiológica. O evento contou com 162 participantes e foi o primeiro grande treinamento presencial realizado durante a epidemia de covid-19 no Brasil. Não foi detectada infecção pelo SARS-CoV-2 ao final do evento em nenhum participante, sugerindo a segurança e efetividade de todas as medidas de segurança adotadas. Os resultados do evento sugerem que é possível executar capacitação profissional com segurança durante pandemias, desde que seguidos todos os protocolos de segurança.

## Resumen

Este estudio tuvo como objetivo describir el primer curso presencial para capacitar a los profesionales de la salud pública para llevar a cabo la vigilancia genómica en tiempo real durante los períodos de pandemia. Este es un informe de experiencia en un curso teórico-práctico centrado en la investigación y vigilancia genómica, que incluye secuenciación móvil, bioinformática, filogenética y tecnologías de modelado epidemiológico. Este evento contó con la asistencia de 162 participantes y fue la primera gran capacitación presencial realizada durante la epidemia de COVID-19 en Brasil. No se detectó infección por SARS-CoV-2 al final del evento en ningún participante, lo que sugiere la seguridad y efectividad de todas las medidas de seguridad adoptadas. Por lo tanto, los resultados del evento sugieren que es posible realizar entrenamientos profesionales de manera segura durante pandemias, siempre y cuando se sigan todos los protocolos de seguridad.

## INTRODUCTION

Pandemics are part of the group of natural disaster events triggered by socio-environmental phenomena and imbalances that affect populations. In this context, on January 30, 2020, the World Health Organization (WHO) characterized the outbreak of Coronavirus Disease 2019 (COVID-19). Having started in Wuhan, China, as a Public Health Emergency of International Concern,^1^ the disease quickly turned into a major global challenge and in March 2020, it was declared as a pandemic, requiring a rapid health response.^2^

Understanding the spread of potentially pandemic viral infectious diseases is fundamental in order to support public health decisions. An important method of monitoring these diseases is genomic and epidemiological surveillance, which enables the rapid identification of these infectious agents. Since the beginning of the^3^ pandemic, the spread of the causative agent - Severe Acute Respiratory Syndrome Coronavirus 2 (SARS-CoV-2) - of COVID-19 has been monitored worldwide, with different surveillance strategies, by which the virus evolution can be observed and shared in real time.^4^

In Brazil, each Federative Unit (UF), including the Federal District, has a Central Public Health Laboratory (*Laboratório Central de Saúde Pública* - LACEN), under the General Coordination of Public Health Laboratories (*Coordenação-Geral de Laboratórios de Saúde Pública* - CGLAB), within Health and Environment Surveillance Secretariat of the Ministry of Health. The LACENs have, as their main function, to assist the states in the areas of epidemiological, sanitary and environmental surveillance (Organic Law No. 8080).^5^ Consequently, these laboratories are involved in the diagnosis and monitoring of diseases of public health concern and are responsible for a significant portion of the diagnosis of emerging and re-emerging diseases, such as arboviruses and COVID-19.

During the COVID-19 pandemic, LACENs played a key role in identifying variants of concern, given that they receive samples from all regions of the country. However, in addition to the basic infrastructure, updating the bioinformatics tools necessary for data analysis is extremely important. This analysis still represents a major challenge for public health professionals, since it involves specific techniques from the area of scientific research and that are not widely used in practical experience at LACENs.

In an attempt to strengthen the action of national epidemiological surveillance through technology transfer, in September 2021, the first face-to-face training was held in Brazil, where the epidemiological and genomic data generated were analyzed by health professionals from LACENs, during the event entitled Bioinformatics Workshop on Virus Evolution and Molecular Epidemiology (VEME).

VEME is a traditional course aimed at training participants in the use of bioinformatics tools applied to epidemiology and virus evolution. It features internationally acclaimed professors who are expert on that field, and its founder and organizer is Professor Anne-Mieke Vandamme from KU Leuven, University of Leuven, Belgium. This is an annual event with more than 26 editions, each of them is held in a different city in the world.^6^ The event’s basic premise is the dissemination of scientific knowledge, providing quality training and capacity building, as well as technology transfer to students and professionals from all continents.

With the support of the Pan American Health Organization/World Health Organization (PAHO/WHO), the National Council for Scientific and Technological Development (*Conselho Nacional de Desenvolvimento Científico e Tecnológico* - CNPq) and the Health Surveillance Secretariat/Ministry of Health (SVS/MS), VEME was held in Brazil, in September 2021.^6^

This experience report aims to discuss a face-to-face theoretical and practical course, conducted during the COVID-19 pandemic, for public health professionals, focusing on genomic research and surveillance. The course included topics such as mobile sequencing technologies, bioinformatics, phylogenetics and epidemiological modeling.

## METHODS

Traditionally, the previous 26 editions of VEME took place in a face-to-face format. Due to the COVID-19 pandemic in 2021, VEME had two versions: (i) COVEME, a webinar format event focused on the application of bioinformatics tools in corona virus research; and (ii) VEME light, a face-to-face event focused on training and technology transfer. The event was conducted in both formats based on the concept of Responsible Research and Innovation (RRI), which aims to strengthen the interactions between science and society.^7^ ^),(^ ^8^

As a didactic strategy for training and updating health professionals enrolled in the course, the most commonly used bioinformatics tools in high-impact research were selected: Genome Detective for genome assembly; MAFFT for sequence alignment; AliView for editing sequences manually; IQ-TREE, for estimating phylogenetic signal and constructing maximum likelihood trees; TempEst for estimating the temporal signal; BEAST for constructing dated trees; FigTree for visualizing generated phylogenetic trees; and R Studio for epidemiological analyses and data visualization.^9^ ^)-(^ ^16^

VEME light took place between September 5 and 10, 2021, at Ouro Minas Palace Hotel, Belo Horizonte, state of Minas Gerais, Brazil, according to the activity program described in Box 1. In order to ensure the safety of all participants, the event management team established the sanitary protocols to be followed.

**Box 1:** Description of the activities performed during VEME light

Sunday, September 5
Registration and Accreditation + sample collection for SARS-CoV-2 diagnosis
Linux command line
Panorama and Surveillance of COVID-19 in Brazil and Latin America Panorama and Surveillance of Arboviruses in Brazil
Yellow Fever, Mayaro, Oropouche and Mayaro viruses in the Brazilian North and Northeast regions Zika, Dengue and Chikungunya viruses in the Brazilian Southeast and Northeast regions SARS-CoV-2 in Brazil
Zoonoses and the Emergence of COVID-19
Monday, September 6
Linux command line
Reference-based genome assembly and error correction algorithms
Real time genomic surveillance of SARS-CoV-2 in South Africa
De novo genome assembly using Genome Detective software
Tuesday, September 7
Constructing a proper dataset using NCBI web-based tools
Alignment algorithms and Aliview
COVID-19: how sharing, communication and Nextstrain have played a part in the outbreak
IQ-TREE
Alignment and Phylogenetic signal
Wednesday, September 8
Methods for phylogenetic tree reconstruction (NJ e ML)
TempEST
Predicting mosquito-borne transmission using climate data
IQ-TREE e TempEST
Thursday, September 9
Molecular clocks (applications + TreeTime)
TreeTime and BEAST
Friday, September 10
Rstudio
Sample collection for SARS-CoV-2 diagnosis

All participants, professors and organizers stayed in single rooms in the hotel where the event took place. The hotel offered a buffet service, with alcohol-based hand sanitizer available in order for all of them to use before putting on gloves (to serve themselves) and settling down in a room specially organized for the participants.

The course activities were carried out in a room named Salão Centenário, which was capable of accommodating 900 people, with tables arranged to maintain the necessary social distancing. Each student received a seat and a specific computer, for in silico practices. It was not allowed to change the equipment during the event. The seats and computers were arranged to ensure a minimum distance of 2 meters between them. The hotel cleaning staff performed daily disinfection of the computers and tables using 70% isopropyl alcohol. Water was served in an area outside the classroom in order to ensure the necessary distancing.

Students and professors were provided with face shield and an adequate supply of NK95/PFF2 masks that allowed their exchanged every five hours. The use of the personal protective equipment (PPE) was mandatory throughout the course, with a constant reminder to use 70% alcohol-based hand sanitizer.

Everyone was advised not to leave the hotel during the days of the event.

The training participants, organizing committee, professors and hotel staff were tested for SARS-CoV-2 before the start and at the end of the event. Thus, nasopharyngeal samples were collected and the polymerase chain reaction (PCR) test was performed by employees of Fundação Ezequiel Dias (LACEN/MG), in an equipped temporary room at the event venue. PCR test was performed using Allplex 2019-nCoV Assay (Seegene), aiming to detect the envelope (E), the viral RNA-dependent RNA-polymerase (RdRp) or RNA replicase and the nucleocapsid (N) gene.

## RESULTS

VEME light had a total of 162 participants, including 146 students and 16 professors. Among the students, 131 came from 21 different Brazilian institutions: 64 employees of LACENs and CGLAB; and 67 came from other municipal, academic and research institutions directly involved in emerging and re-emerging infections. The remaining 15 students were from other countries: Mexico, Paraguay, Uruguay, Guatemala, the Dominican Republic and Senegal.

For the selection of students, gender equity was taken into account, in addition to the participation of two to three representatives of each of the 27 LACENs in the country. These professionals work directly with the sequencing of SARS-CoV-2 and data analysis. The training course enabled them to perform the necessary analyses for genomic surveillance (genome assembly, phylogenetic analyses, identification of viral variants) and as a result, several scientific publications authored by these professionals are currently underway.^17^ ^)-(^ ^20^

**Figure 1:**
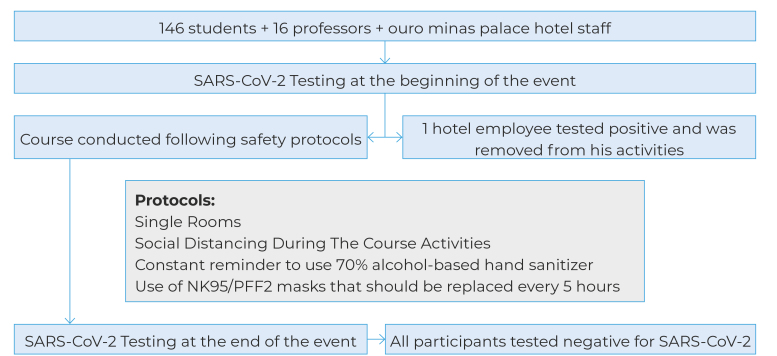
VEME light flowchart with description of safety protocols

All 162 participants, as well as Ouro Minas Palace Hotel staff, were tested for SARS-CoV-2 before the start of the event. Only one hotel employee tested positive and was removed from his activities; among the other participants and hotel staff, no SARS-CoV-2 infection was detected, neither before nor at the end of the event, indicating the safety and effectiveness of all the measures adopted (Figure 1).

## DISCUSSION

In recent years, at the initiative of the Ministry of Health, the LACENs have been implementing different Next-Generation Sequencing (NGS) strategies to perform genomic surveillance. This process has enabled each LACEN to generate complete genomes of emerging and re-emerging viral pathogens, circulating and co-circulating in each state. In this scenario, the need arose to train healthcare workers in the use of bioinformatics tools, enabling them to analyze genomic data generated in their laboratories in real time. This would have facilitated the rapid dissemination - among public health authorities - of the number of genotypes and subtypes of the circulating viral strains detected by each of the 27 LACENs, so that control measures could be promptly implemented. Given the demand, this training course has initiated the technology transfer process and capacity building of public health professionals in Brazil for the real-time monitoring and analysis of genomic data.

It is worth highlighting that during VEME light, concepts of Responsible Research and Innovation RRI were addressed. It is a movement that aims to restructure the way science is integrated into society.^7^ ^),(^ ^8^ In Brazil, the RRI acronym is not widely adopted and promoted; however, many everyday practices are in line with the spirit of RRI, which seeks a more responsive, reflective, inclusive, and socially responsible science and technology.

The concept of RRI involves five key principles: Education; Open Access; Ethics; Social Engagement; Gender Equality.^7^ ^),(^ ^8^ These principles were put into practice during the training course, with education remaining the core of VEME.

Education is the basis for technology transfer and capacity building, enabling the dissemination and application of knowledge.^21^ During VEME, in addition to the participation of professors who are experts on that field and internationally recognized for their work, the classes were based on a practical learning approach.

The didactic strategy used ensured that the students could apply the tools in real time and that the content covered was more suitable for the practice of each LACEN. Furthermore, in order for each student and public health professional to master the tools presented, they were granted autonomy to apply and disseminate the knowledge acquired at the face-to-face VEME, in their state.

VEME provided public health professionals from all Brazilian states with access to knowledge and tools used by leading research centers in the world. This training course has enabled faster detection and monitoring of circulating viral variants in the states, given that trained health professionals began performing real-time genomic surveillance and publishing their results in high-impact journals.^17^ ^)-(^ ^20^

The fact that the event organizers only advised the participants to stay at the hotel during the days of the event, without obligating them, and that there was no control over their entry and exit, is a limitation of this study. However, the spirit of collaboration of the professors and enrolled students was reflected in everyone staying at the training location, respecting the proposed safety protocols.

The implementation of technology transfer in a face-to-face course, during the COVID-19 epidemic in Brazil, was of great relevance for the training of public health professionals involved. The topics covered during the event enabled not only genomic data generation, but also their rapid analysis. This training process was fundamental for monitoring the evolution of the epidemic at the local level. Motivated by the success of this experience, a new module entitled Virus Evolution to Public Health Policy Makers will be incorporated into the next edition of VEME in Panama in 2022. This training course for health professionals in genomic data analysis represents a milestone in epidemiological surveillance, allowing Brazil not only to efficiently monitor endemic strains, but also to predict new outbreaks through active monitoring and real-time data analysis.
